# Supplementary material for: Multi-domain interaction mediated strength-building in human α-actinin dimers unveiled by direct single-molecule quantification
Source: Nat Commun. 2024 Jul 21;15:6151. doi: 10.1038/s41467-024-50430-w (PMC11271494; doi:10.1038/s41467-024-50430-w)
Supplement: Supplementary file 1 — Supplementary Information [file 41467_2024_50430_MOESM1_ESM.pdf]

Supplementary Information for
Multi-domain Interaction Mediated Strength-Building in Human α-Actinin Dimers Unveiled by Direct Single-molecule Quantification

Contents

1 Supplementary Note **2**

1.1 Supplementary Note S1.
Detailed sequence information of the single-molecule protein constructs 2

2 Supplementary Figures **5**

2.1 Supplementary Figure S1.
Illustration of the single-molecule tethering strategies. 5

2.2 Supplementary Figure S2.
Illustration of bead rotation due to torque rebalance in magnetic tweezers experiments. . . 6

2.3 Supplementary Figure S3.
Theoretical force-dependent rupture or unfolding transition step sizes of α-actinin dimers and domains 7

2.4 Supplementary Figure S4.
Example force–bead height curves of the α-actinin 1 homo-dimer under shear-force geometry during force-loading experiments. 8

2.5 Supplementary Figure S5.
Rupture and re-dimerization of the α-actinin 1 homo-dimer under shear-force geometry with or without DNA handle in the tethers 9

2.6 Supplementary Figure S6.
Time-dependent rupture probability analysis of α-actinin rod dimers 10

2.7 Supplementary Figure S7.
Time-dependent rupture probability analysis of α-actinin 1 SR2-SR3-SR4:SR1-SR2-SR3 dimer 11

2.8 Supplementary Figure S8.
Time-dependent rupture probability analysis of *Entamoeba histolytica* α-actinin 2 dimer . . 12

2.9 Supplementary Figure S9.
High mechanical stabiliy of the human α-actinin 1 &4 hetero-dimer under shear-stretching force geometry 13

2.10 Supplementary Figure S10.
Interconversion of the mechanical states of the α-actinin 1 SR2-SR3-SR4:SR1-SR2-SR3 dimer at a constant force 14

2.11 Supplementary Figure S11.
Force-response of α-actinin 1 SR2-SR3-SR4 domains 15

2.12 Supplementary Figure S12.
The representative force–bead height curves of the α-actinin 1 SR1:SR4 pair 16

3 Supplementary References **16**

1 Supplementary Note

1.1 Supplementary Note S1. Detailed sequence information of the single-molecule protein constructs

Eight plasmids were prepared for expression of the protein constructs for single-molecule stretching experiments,and a Spycatcher003-containing plasmid were prepared for expression of the Spycatcher protein with a C-terminal cysteine residue.

1. pET151- Spy003- (SR1-SR2-SR3-SR4)$_{\text{ACTN1}}$- (GS Linker)- AviTag- (SR1-SR2-SR3-SR4)$_{\text{ACTN1}}$
2. pET151- Spy003- (SR1-SR2-SR3-SR4)$_{\text{ACTN4}}$- (GS Linker)- AviTag- (SR1-SR2-SR3-SR4)$_{\text{ACTN1}}$
3. pET151- Spy003- (SR1-SR2-SR3-SR4)$_{\text{ACTN1}}$- (GS Linker)- AviTag- (SR1-SR2-SR3-SR4)$_{\text{ACTN4}}$
4. pET151- AviTag- (SR1-SR2-SR3-SR4)$_{\text{ACTN1}}$- (Long Linker)- (SR1-SR2-SR3-SR4)$_{\text{ACTN1}}$- Spy
5. pET151- Spy003- (SR2-SR3-SR4)$_{\text{ACTN1}}$- (GS Linker)- AviTag- (SR1-SR2-SR3)$_{\text{ACTN1}}$
6. pET151- Spy003- (SR2-SR3)$_{\text{ACTN1}}$- (GS Linker)- AviTag- (SR2-SR3)$_{\text{ACTN1}}$
7. pET151- Spy003- (SR1)$_{\text{ACTN1}}$- (GS Linker)- AviTag- (SR4)$_{\text{ACTN1}}$
8. pET151- Spy003- (SR1-SR2)$_{\text{Entamoeba histolytica}}$- (GS Linker)- AviTag- (SR1-SR2)$_{\text{Entamoeba histolytica}}$
9. pET151- Spycatcher003-Cys

The detailed sequence information of the corresponding seven protein constructs are listed below:

1. Spy003- (SR1-SR2-SR3-SR4)$_{\text{ACTN1}}$- (GS Linker)-AviTag- (SR1-SR2-SR3-SR4)$_{\text{ACTN1}}$:
   MRGVPHIVMVDAYKRYKGGGSGGGGSGKLGGGKVLAVNQENEQLMEDYEKLASDLLEWI
   RRTIPWLENRVPENTMHAMQQKLEDFRDYRRLHKPPKVQEKCQLEINFNTLQTKLRLSNRPAF
   MPSEGRMVSDINNAWGCLEQVEKGYEEWLLNEIRRLERLDHLAEKFRQKASIHEAWTDGKEA
   MLRQKDYETATLSEIKALLKKHEAFESDLAAHQDRVEQIAAIAQELNELDYYDSPSVNARCQKI
   CDQWDNLGALTQKRREALERTEKLLETIDQLYLEYAKRAAPFNNWMEGAMEDLQDTFIVHTI
   EEIQGLTTAHEQFKATLPDADKERLAILGIHNEVSKIVQTYHVNMAGTNPYTTITPQEINGKWD
   HVRQLVPRRDQALTEEHARQQHNERLRKQFGAQANVIGPWIQTKMEEIGRISIEMHGTLEDQL
   SHLRQYEKSIVNYKPKIDQLEGDHQLIQEALIFDNKHTNYTMEHIRVGWEQLLTTIARTINEVEN
   QILTRDAKGISQGGGSGEFGGGSGGGGTGGGSPWGGSGGSGSGSGSGSGGSGSGSGSGSGGGS
   GGPGGSGGGGSGGGGSTGGGSGGGGSGGSSGSGSSGSGSSGSSGSSGSGSGGSGSSGSGGGGGS
   SGSGSSGSSGSGSGGGSGSGSSGGGSGGSGSGSGSGSVDGGGSGGGLNDIFEAQKIEWHESGGSG
   GSSGKVLAVNQENEQLMEDYEKLASDLLEWIRRTIPWLENRVPENTMHAMQQKLEDFRDYRR
   LHKPPKVQEKCQLEINFNTLQTKLRLSNRPAFMPSEGRMVSDINNAWGCLEQVEKGYEEWLLN
   EIRRLERLDHLAEKFRQKASIHEAWTDGKEAMLRQKDYETATLSEIKALLKKHEAFESDLAAHQ
   DRVEQIAAIAQELNELDYYDSPSVNARCQKICDQWDNLGALTQKRREALERTEKLLETIDQLYL
   EYAKRAAPFNNWMEGAMEDLQDTFIVHTIEEIQGLTTAHEQFKATLPDADKERLAILGIHNEVS
   KIVQTYHVNMAGTNPYTTITPQEINGKWDHVRQLVPRRDQALTEEHARQQHNERLRKQFGAQ
   ANVIGPWIQTKMEEIGRISIEMHGTLEDQLSHLRQYEKSIVNYKPKIDQLEGDHQLIQEALIFDNK
   HTNYTMEHIRVGWEQLLTTIARTINEVENQILTRDAKGISQGSGLEGGGSGKLGDIEFIKVNKG
   GGSGENLYFQGHHHHHH*
2. Spy003- (SR1-SR2-SR3-SR4)$_{\text{ACTN4}}$- (GS Linker)-AviTag- (SR1-SR2-SR3-SR4)$_{\text{ACTN4}}$:
   MRGVPHIVMVDAYKRYKGGGSGGGGSGKLGGGKVLAVNQENEHLMEDYEKLASDLLEWI
   RRTIPWLEDRVPQKTIQEMQQKLEDFRDYRRVHKPPKVQEKCQLEINFNTLQTKLRLSNRPAF
   MPSEGKMVSDINNGWQHLEQAEKGYEEWLLNEIRRLERLDHLAEKFRQKASIHEAWTDGKEA
   MLKHRDYETATLSDIKALIRKHEAFESDLAAHQDRVEQIAAIAQELNELDYYDSHNVNTRCQKI
   CDQWDALGSLTHSRREALEKTEKQLEAIDQLHLEYAKRAAPFNNWMESAMEDLQDMFIVHTI
   EEIEGLISAHDQFKSTLPDADREREAILAIHKEAQRIAESNHIKLSGSNPYTTVTPQIINSKWEKV
   QQLVPKRDHALLEEQSKQQSNEHLRRQFASQANVVGPWIQTKMEEIGRISIEMNGTLEDQLSHL
   KQYERSIVDYKPNLDLLEQQHQLIQEALIFDNKHTNYTMEHIRVGWEQLLTTIARTINEVENQIL
   TRDAKGISQGGGSGEFGGGSGGGGTGGGSPWGGSGGSGSGSGSGSGGSGSGSGSGSGGGSGGP
   GGSGGGGSGGGGSTGGGSGGGGSGGSSGSGSSGSGSSGSSGSSGSGSGGSGSSGSGGGGGSSGSG
   SSGSSGSGSGGGSGSGSSGGGSGGSGSGSGSGSVDGGGSGGGLNDIFEAQKIEWHESGGSGGSSG
   KVLAVNQENEHLMEDYEKLASDLLEWIRRTIPWLEDRVPQKTIQEMQQKLEDFRDYRRVHKP
   PKVQEKCQLEINFNTLQTKLRLSNRPAFMPSEGKMVSDINNGWQHLEQAEKGYEEWLLNEIRR
   LERLDHLAEKFRQKASIHEAWTDGKEAMLKHRDYETATLSDIKALIRKHEAFESDLAAHQDRV
   EQIAAIAQELNELDYYDSHNVNTRCQKICDQWDALGSLTHSRREALEKTEKQLEAIDQLHLEYA
   KRAAPFNNWMESAMEDLQDMFIVHTIEEIEGLISAHDQFKSTLPDADREREAILAIHKEAQRIAE
   SNHIKLSGSNPYTTVTPQIINSKWEKVQQLVPKRDHALLEEQSKQQSNEHLRRQFASQANVVGP
   WIQTKMEEIGRISIEMNGTLEDQLSHLKQYERSIVDYKPNLDLLEQQHQLIQEALIFDNKHTNYT

MEHIRVGWEQLLTTIARTINEVENQILTRDAKGISQGSGLEGGGSGKLGDIEFIKVNKGGGSGEN
LYFQGHHHHHH*

3. Spy003- (SR1-SR2-SR3-SR4)$_{\mathrm{ACTN1}}$- (GS Linker)-AviTag- (SR1-SR2-SR3-SR4)$_{\mathrm{ACTN4}}$:

MRGVPHIVMVDAYKRYKGGGSGGGGSGKLGGGKVLAVNQENEQLMEDYEKLASDLLEWI
RRTIPWLENRVPENTMHAMQQKLEDFRDYRRLHKPPKVQEKCQLEINFNTLQTKLRLSNRPAF
MPSEGRMVSDINNAWGCLEQVEKGYEEWLLNEIRRLERLDHLAEKFRQKASIHEAWTDGKEA
MLRQKDYETATLSEIKALLKKHEAFESDLAAHQDRVEQIAAIAQELNELDYYDSPSVNARCQKI
CDQWDNLGALTQKRREALERTEKLLETIDQLYLEYAKRAAPFNNWMEGAMEDLQDTFIVHTI
EEIQGLTTAHEQFKATLPDADKERLAILGIHNEVSKIVQTYHVNMAGTNPYTTITPQEINGKWD
HVRQLVPRRDQALTEEHARQQHNERLRKQFGAQANVIGPWIQTKMEEIGRISIEMHGTLEDQL
SHLRQYEKSIVNYKPKIDQLEGDHQLIQEALIFDNKHTNYTMEHIRVGWEQLLTTIARTINEVEN
QILTRDAKGISQGGGSGEFGGGSGGGGTGGGSPWGGSGGSGSGSGSGGGSGSGSGSGSGGGS
GGPGGSGGGGSGGGGSTGGGSGGGGSGGSSGSGSSGSGSSGSSGSSGSGSGGSGSSGSGGGGGS
SGSGSSGSSGSGSGGGGSGSGSSGGGSGGSGSGSGSGSVDGGGSGGGLNDIFEAQKIEWHESGGSG
GSSGKVLAVNQENEHLMEDYEKLASDLLEWIRRTIPWLEDRVPQKTIQEMQQKLEDFRDYRRV
HKPPKVQEKCQLEINFNTLQTKLRLSNRPAFMPSEGKMVSDINNGWQHLEQAEKGYEEWLLN
EIRRLERLDHLAEKFRQKASIHEAWTDGKEAMLKHRDYETATLSDIKALIRKHEAFESDLAAHQ
DRVEQIAAIAQELNELDYYDSHNVNTRCQKICDQWDALGSLTHSRREALEKTEKQLEAIDQLHL
EYAKRAAPFNNWMESAMEDLQDMFIVHTIEEIEGLISAHDQFKSTLPDADREREAILAIHKEAQ
RIAESNHIKLSGSNPYTTVTPQIINSKWEKVQQLVPKRDHALLEEQSKQQSNEHLRRQFASQAN
VVGPWIQTKMEEIGRISIEMNGTLEDQLSHLKQYERSIVDYKPNLDLLEQQHQLIQEALIFDNKH
TNYTMEHIRVGWEQLLTTIARTINEVENQILTRDAKGISQGSGLEGGGSGKLGDIEFIKVNKGG
GSGENLYFQGHHHHHH*

4. AviTag- (SR1-SR2-SR3-SR4)$_{\mathrm{ACTN1}}$- (FH1-Linker)- (SR1-SR2-SR3-SR4)$_{\mathrm{ACTN1}}$-Spy003:

MHHHHHHGKPIPNPLLGLDSTENLYFQGIDPFTGLNDIFEAQKIEWHEGGTGGTGGTAGCG
GTAAGCTTAFSGAQKAETAANRICKVLAVNQENEQLMEDYEKLASDLLEWIRRTIPWLENRVP
ENTMHAMQQKLEDFRDYRRLHKPPKVQEKCQLEINFNTLQTKLRLSNRPAFMPSEGRMVSDIN
NAWGCLEQVEKGYEEWLLNEIRRLERLDHLAEKFRQKASIHEAWTDGKEAMLRQKDYETATL
SEIKALLKKHEAFESDLAAHQDRVEQIAAIAQELNELDYYDSPSVNARCQKICDQWDNLGALTQ
KRREALERTEKLLETIDQLYLEYAKRAAPFNNWMEGAMEDLQDTFIVHTIEEIQGLTTAHEQF
KATLPDADKERLAILGIHNEVSKIVQTYHVNMAGTNPYTTITPQEINGKWDHVRQLVPRRDQA
LTEEHARQQHNERLRKQFGAQANVIGPWIQTKMEEIGRISIEMHGTLEDQLSHLRQYEKSIVNY
KPKIDQLEGDHQLIQEALIFDNKHTNYTMEHIRVGWEQLLTTIARTINEVENQILTRDAKGISQL
EPPAPPLPGDSGTIIPPPPAPGDSTTPPPPPPPPPPPPPLPGGVCISSPPSLPGGTAISPPPPLSGD
ATIPPPPPLPEGVGIPSPSSLPGGTAIPPPPPLPGSARIPPPPPPLPGSAGIPPPPPPLPGEAGMPP
PPPPLPGGPGIPPPPPFPGGPGIPPPPPGMGMPPPPPFGFGVPAAPVLPLKGFEIDKVWYDLD
AKLGDIEFIKVNKGGGSGAFSGAQKAETAANRICKVLAVNQENEQLMEDYEKLASDLLEWIRR
TIPWLENRVPENTMHAMQQKLEDFRDYRRLHKPPKVQEKCQLEINFNTLQTKLRLSNRPAFM
PSEGRMVSDINNAWGCLEQVEKGYEEWLLNEIRRLERLDHLAEKFRQKASIHEAWTDGKEAM
LRQKDYETATLSEIKALLKKHEAFESDLAAHQDRVEQIAAIAQELNELDYYDSPSVNARCQKIC
DQWDNLGALTQKRREALERTEKLLETIDQLYLEYAKRAAPFNNWMEGAMEDLQDTFIVHTIE
EIQGLTTAHEQFKATLPDADKERLAILGIHNEVSKIVQTYHVNMAGTNPYTTITPQEINGKWDH
VRQLVPRRDQALTEEHARQQHNERLRKQFGAQANVIGPWIQTKMEEIGRISIEMHGTLEDQLS
HLRQYEKSIVNYKPKIDQLEGDHQLIQEALIFDNKHTNYTMEHIRVGWEQLLTTIARTINEVENQ
ILTRDAKGISQEFGGGSGAHIVMVDAYKPTK*

5. Spy003- (SR2-SR3-SR4)$_{\mathrm{ACTN1}}$- (GS Linker)-AviTag- (SR1-SR2-SR3)$_{\mathrm{ACTN1}}$:

MRGVPHIVMVDAYKRYKGGGSGGGGSGKLGGEIRRLERLDHLAEKFRQKASIHEAWTDGK
EAMLRQKDYETATLSEIKALLKKHEAFESDLAAHQDRVEQIAAIAQELNELDYYDSPSVNARCQ
KICDQWDNLGALTQKRREALERTEKLLETIDQLYLEYAKRAAPFNNWMEGAMEDLQDTFIVH
TIEEIQGLTTAHEQFKATLPDADKERLAILGIHNEVSKIVQTYHVNMAGTNPYTTITPQEINGK
WDHVRQLVPRRDQALTEEHARQQHNERLRKQFGAQANVIGPWIQTKMEEIGRISIEMHGTLED
QLSHLRQYEKSIVNYKPKIDQLEGDHQLIQEALIFDNKHTNYTMEHIRVGWEQLLTTIARTINEV
ENQILTRDAKGISQGGGSGEFGGGSGGGGTGGGSPWGGSGGSGSGSGSGGSGSGSGSGG
GSGGPGGSGGGGSGGGGSTGGGSGGGGSGGSSGSGSSGSGSSGSSGSSGSGSGGSGSSGSGGGG
GSSGSGSSGSSGSGSGGGSGSGSSGGGSGGSGSGSGSGSVDGGGSGGGLNDIFEAQKIEWHESGG
SGGSSGKVLAVNQENEQLMEDYEKLASDLLEWIRRTIPWLENRVPENTMHAMQQKLEDFRDY
RRLHKPPKVQEKCQLEINFNTLQTKLRLSNRPAFMPSEGRMVSDINNAWGCLEQVEKGYEEW
LLNEIRRLERLDHLAEKFRQKASIHEAWTDGKEAMLRQKDYETATLSEIKALLKKHEAFESDLA
AHQDRVEQIAAIAQELNELDYYDSPSVNARCQKICDQWDNLGALTQKRREALERTEKLLETIDQ
LYLEYAKRAAPFNNWMEGAMEDLQDTFIVHTIEEIQGLTTAHEQFKATLPDADKERLAILGIHN

EVSKIVQTYHVNMAGTNPYTTITPQEINGKWDHVRQLVPRRDQALTEEHARQQHNEGSGLEG
GGSGKLGDIEFIKVNKGGGSGENLYFQGHHHHHH*

6. Spy003- (SR2-SR3)$_{\mathrm{ACTN1}}$- (GS Linker)-AviTag- (SR2-SR3)$_{\mathrm{ACTN1}}$:
MRGVPHIVMVDAYKRYKGGGSGGGGSGKLGGEIRRLERLDHLAEKFRQKASIHEAWTDGK
EAMLRQKDYETATLSEIKALLKKHEAFESDLAAHQDRVEQIAAIAQELNELDYYDSPSVNARCQ
KICDQWDNLGALTQKRREALERTEKLLETIDQLYLEYAKRAAPFNNWMEGAMEDLQDTFIVH
TIEEIQGLTTAHEQFKATLPDADKERLAILGIHNEVSKIVQTYHVNMAGTNPYTTITPQEINGK
WDHVRQLVPRRDQALTEEHARQQHNEGGGSGEFGGGSGGGGTGGGSPWGGSGGSGSGSGSG
SGGSGSGSGSGSGGGSGGPGGSGGGGSGGGGSTGGGSGGGGSGGSSGSGSSGSGSSGSSGSSGS
GSGGSGSSGSGGGGGGSSGSGSSGSSGSGSGGGSGSGSSGGGSGGSGSGSGSGSVDGGGSGGGLND
IFEAQKIEWHESGGSGGSSGEIRRLERLDHLAEKFRQKASIHEAWTDGKEAMLRQKDYETATLS
EIKALLKKHEAFESDLAAHQDRVEQIAAIAQELNELDYYDSPSVNARCQKICDQWDNLGALTQK
RREALERTEKLLETIDQLYLEYAKRAAPFNNWMEGAMEDLQDTFIVHTIEEIQGLTTAHEQFK
ATLPDADKERLAILGIHNEVSKIVQTYHVNMAGTNPYTTITPQEINGKWDHVRQLVPRRDQAL
TEEHARQQHNEGSGLEGGGSGKLGDIEFIKVNKGGGSGENLYFQGHHHHHH*

7. Spy003- (SR1)$_{\mathrm{ACTN1}}$- (GS Linker)-AviTag- (SR4)$_{\mathrm{ACTN1}}$:
MRGVPHIVMVDAYKRYKGGGSGGGGSGKLGGGKVLAVNQENEQLMEDYEKLASDLLEWI
RRTIPWLENRVPENTMHAMQQKLEDFRDYRRLHKPPKVQEKCQLEINFNTLQTKLRLSNRPA
FMPSEGRMVSDINNAWGCLEQVEKGYEEWLLNEIRRLERLDGGGSGEFGGGSGGGGTGGGSP
WGGSGGSGSGSGSGGSGSGSGSGGGSGGPGGSGGGGSGGGGSTGGGSGGGGSGGSSGSG
SSGSGSSGSSGSSGSGSGGSGSSGSGGGGGGSSGSGSSGSSGSGSGGGSGSGSSGGGSGGSGSGSGSG
SVDGGGSGGGLNDIFEAQKIEWHESGGSEHARQQHNERLRKQFGAQANVIGPWIQTKMEEIGR
ISIEMHGTLEDQLSHLRQYEKSIVNYKPKIDQLEGDHQLIQEALIFDNKHTNYTMEHIRVGWEQL
LTTIARTINEVENQILTRDAKGISQGSGLEGGGSGKLGDIEFIKVNKGGGSGENLYFQGHHHHHH
*

8. Spy003- (SR1-SR2)$_{\mathrm{ehACTN2}}$- (GS Linker)-AviTag- (SR1-SR2)$_{\mathrm{ehACTN2}}$:
MRGVPHIVMVDAYKRYKGGGSGGGGSGKLGGGPAMRAGNFLDFLRATEGMVHDYEQRA
QALKENIEAAINKMNGVEPSDEYHQVKEQINETKNYRKGDKRAFIKEQGDLATLFGQINSKLRG
MKRPVYVAPEGLDPKSLEGYIANISEAERALRSKLNTAMRNCLIALRKAFADPANATDAKINEY
RTFVTDETSEAPLEEQVATLKAKLEELKQVEAQLPPIEEAEKACEDANIEDNEYTDVSFDDLQF
NYEQTVSMFEKKIVYIEAQINEASSGGSGEFGGGSGGGGTGGGSPWGGSGGSGSGSGSGGSG
SGSGSGSGGGSGGPGGSGGGGSGGGGSTGGGSGGGGSGGSSGSGSSGSGSSGSSGSSGSGSGGS
GSSGSGGGGGGSSGSGSSGSSGSGSGGGSGSGSSGGGSGGSGSGSGSGSVDGGGSGGGLNDIFEAQ
KIEWHESGGSGGSSGPAMRAGNFLDFLRATEGMVHDYEQRAQALKENIEAAINKMNGVEPSDE
YHQVKEQINETKNYRKGDKRAFIKEQGDLATLFGQINSKLRGMKRPVYVAPEGLDPKSLEGYI
ANISEAERALRSKLNTAMRNCLIALRKAFADPANATDAKINEYRTFVTDETSEAPLEEQVATLK
AKLEELKQVEAQLPPIEEAEKACEDANIEDNEYTDVSFDDLQFNYEQTVSMFEKKIVYIEAQINE
ASSGGSGLEGGGSGKLGDIEFIKVNKGGGSGENLYFQGHHHHHH*

9. SpyCatcher003-Cys:
MHHHHHHGGGGSGVDKLGGSLELKGGGSGAMVTTLSGLSGEQGPSGDMTTEEDSATHIKF
SKRDEDGRELAGATMELRDSSGKTISTWISDGHVKDFYLYPGKYTFVETAAPDGYEVATPIEF
TVNEDGQVTVDGEATEGDAHTGGGSGGSGSGSGSGSGEFGSGGSSGSGSGC*

2 Supplementary Figures

2.1 Supplementary Figure S1. Illustration of the single-molecule tethering strategies.

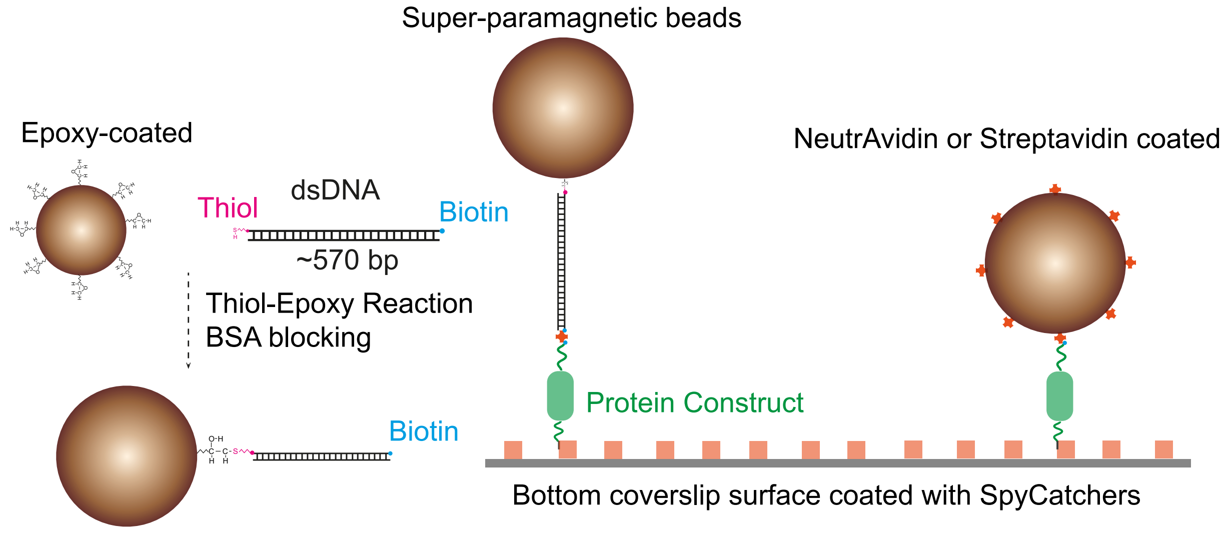

Supplementary Figure S1. Illustration of the single-molecule tethering strategies. Left panel: The key step for preparing the superparamagnetic beads with DNA handles via epoxy-thiol reaction. Middle panel: the illustration of a single target protein tethered between a Spycatcher003-coated coverslip surface and a biotinylated DNA handle on a superparamagnetic bead. Right panel: the illustration of a single target protein tethered between a Spycatcher003-coated coverslip surface and a streptavidin- or neutravidin- coated superparamagnetic bead. Utilizing the characterized overstretching signal of DNA at ∼65 pN, where the extension of the DNA elongated for ∼1.7 times[1, 2], a short DNA of several hundreds of base-pairs is often used in single-molecule stretching experiments as a positive control. In this study, a ∼570 bp DNA handle was used for initial test (as shown in Fig. S5). Since the rupture signal of the rod dimer also occurs at >60 pN, we mainly use the tethering strategy without DNA (right panel).

2.2 Supplementary Figure S2. Illustration of bead rotation due to torque rebalance in magnetic tweezers experiments.

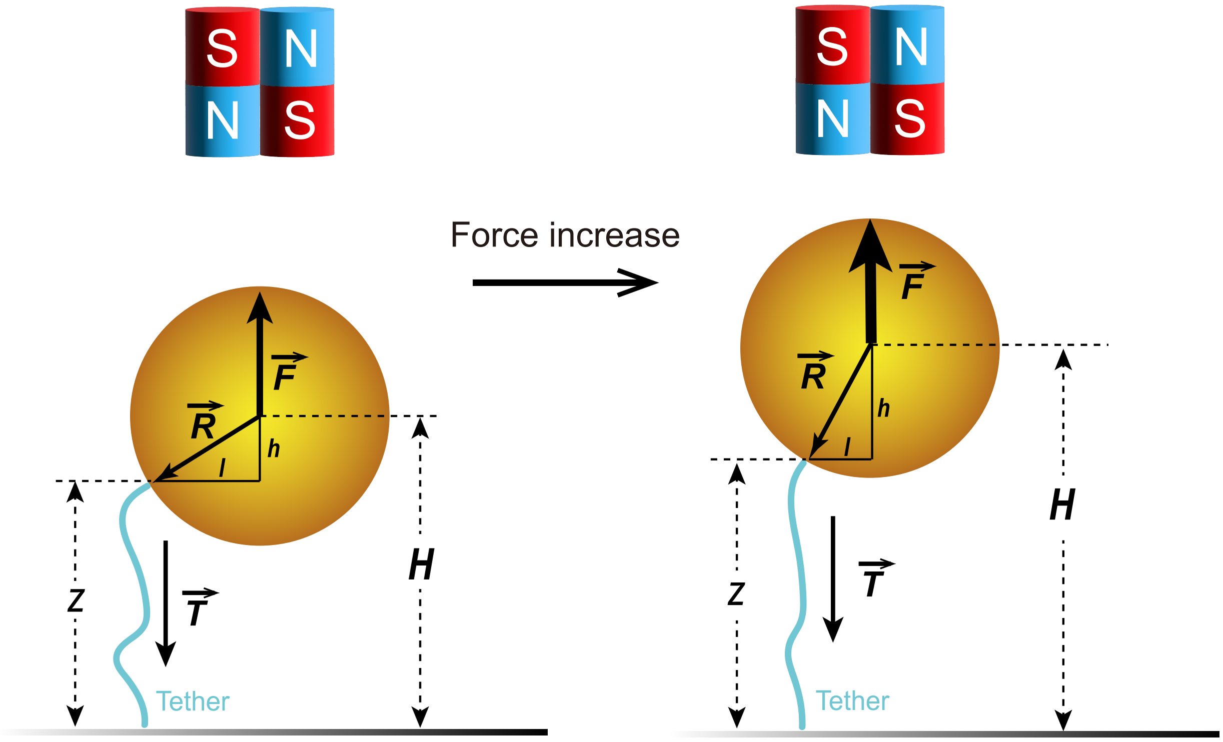

$$\tau_{\text{tension}} = \vec{F} \times \vec{R}$$

Supplementary Figure S2. Illustration of bead rotation due to torque rebalance in magnetic tweezers experiments. In typical magnetic tweezers setup, the superparamagnetic bead has at least one anisotropic magnetization axis of the bead, which is often slightly tilted from the magnetic field due to the heterogeneity of the magnetization of the bead. When applying the magnetic field to the molecule tethered superparamagnetic bead, the alignment of the anisotropic magnetization axis of the bead leads to bead re-orientation, resulting in the typical off-center tethering of the molecule to the bead. Hence, the bead height (H) depends on both the extension of the molecule (z) and off-center tethering due to the torque balance (h). When applied force changes, in order to balance the corresponding force-induced torque changes, the bead rotate accordingly. The rotation of the bead introduces additional bead height change. More details of the off-center tethering could be found in a review on magnetic tweezer experiments[3].

2.3 Supplementary Figure S3. Theoretical force-dependent rupture or unfolding transition step sizes of α-actinin dimers and domains

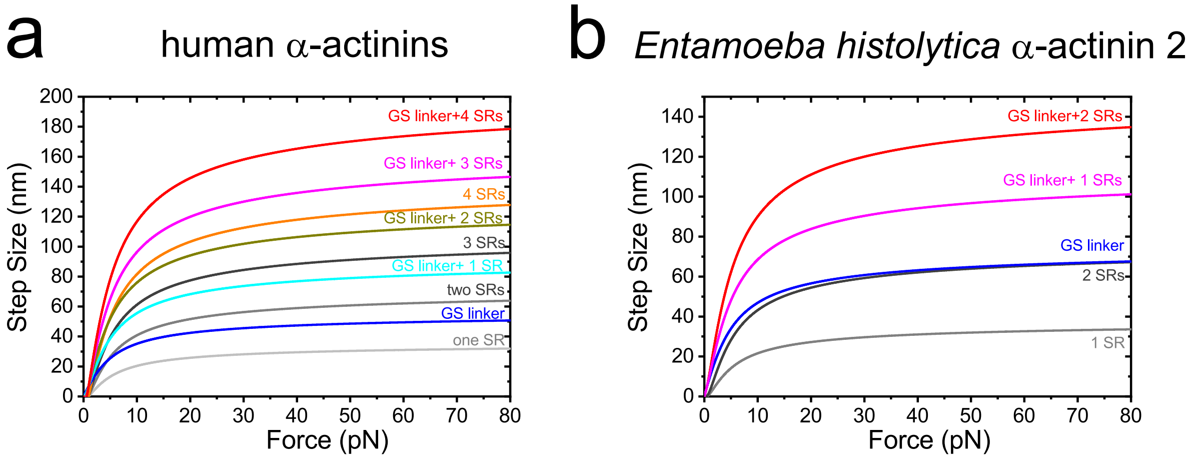

Supplementary Figure S3. Theoretical force-dependent rupture or unfolding transition step sizes of α-actinin dimer and domains. (a). Theoretical force-dependent rupture or unfolding transition step sizes of human α-actinin dimer and domains including scenarios of 1) one SR domain unfolding (light gray), two SR domain concurrent unfolding (gray), three SR domains concurrent unfolding (dark gray), four SR domains concurrent unfolding (orange), GS linker releasing upon dimer rupture (blue), GS linker releasing and concurrent unfolding of one SR domain upon dimer rupture (cyan), GS linker releasing and concurrent unfolding of two SR domains upon dimer rupture (dark yellow), GS linker releasing and concurrent unfolding of three SR domains upon dimer rupture (magenta), and GS linker releasing and concurrent unfolding of four SR domains (the full rod) upon dimer rupture (red). The GS linker used for human α-actinin dimers in shear-force geometry contains ∼160 residues. **(b)**. Theoretical force-dependent rupture or unfolding transition step sizes of *Entamoeba histolytica* α-actinin 2 dimer and domains including scenarios of 1) one SR domain unfolding (light gray), two SR domain concurrent unfolding (dark gray), GS linker releasing upon dimer rupture (blue), GS linker releasing and concurrent unfolding of one SR domain upon dimer rupture (magenta), and GS linker releasing and concurrent unfolding of two SR domains (the full rod) upon dimer rupture (red). The GS linker used for *Entamoeba histolytica* α-actinin 2 dimer contains ∼200 residues. Source data are provided as a Source Data file.

2.4 Supplementary Figure S4. Example force–bead height curves of the α-actinin 1 homo-dimer under shear-force geometry during force-loading experiments.

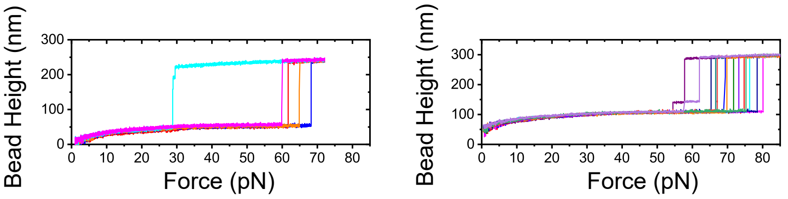

Supplementary Figure S4. Example force–bead height curves of the α-actinin 1 homo-dimer under shear-force geometry during force-loading experiments. Representative force–bead height curves of the α-actinin 1 homo-dimer under shear-force geometry during linear force-loading experiments, obtained from two other molecules (Left and Right). Source data are provided as a Source Data file.

2.5 Supplementary Figure S5. Rupture and re-dimerization of the α-actinin 1 homo-dimer under shear-force geometry with or without DNA handle in the tethers

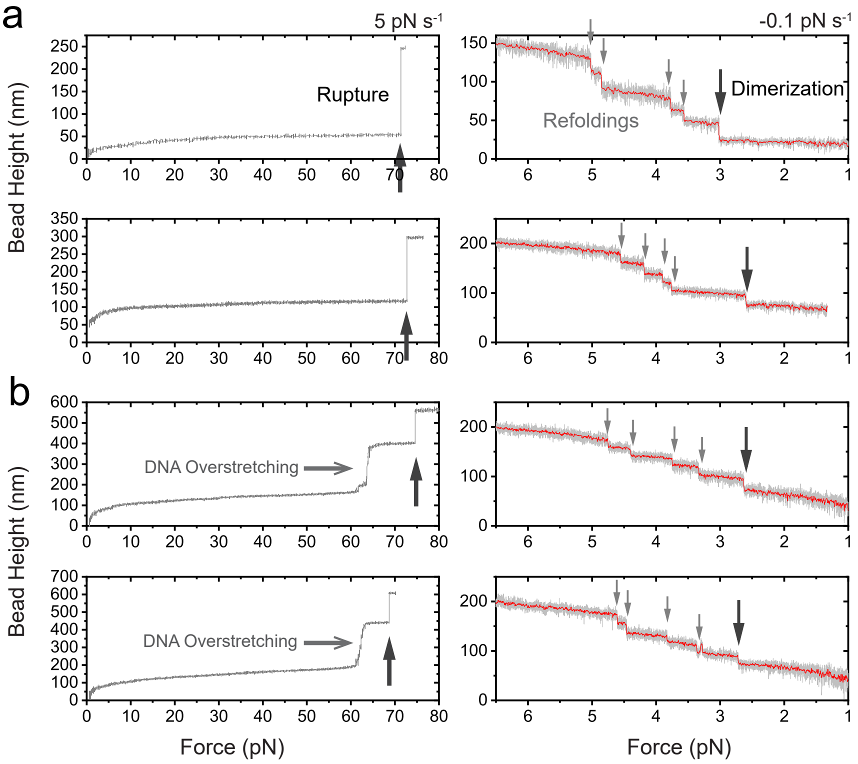

Supplementary Figure S5. Rupture and re-dimerization of the α-actinin 1 homo-dimer under shear-force geometry with or without DNA handle in the tethers. (a). Left panel: Two representative force–bead height curves of the α-actinin 1 homo-dimer construct under shear-force geometry without DNA handle during a linear force-increase scan with a loading rate of 5 pN s^{-1}, the black up-arrows indicate the concurrent rupturing and unfolding event. Right panel: Two representative force–bead height curves of the α-actinin 1 homo-dimer re-folding and re-dimerization during a linear force-decrease scan with a loading rate of -0.1 pN s^{-1} after rupturing and unfolding during force-increase scans as shown in left panel. For the right panels, the gray down-arrows indicate the re-folding events, and black the down-arrows indicate the re-dimerization events. The characteristic SR refolding signals at 3-6 pN forces could act as positive controls for molecule specificity. **(b)**. Left panel: Two representative force–bead height curves of the α-actinin 1 homo-dimer construct under shear-force geometry with DNA handle during a linear force-increase scan with a loading rate of 5 pN s^{-1}, the black up-arrows indicate the concurrent rupturing and unfolding events, the gray right-arrows indicate the DNA overstretching event at $\sim$65 pN. A specific characteristic DNA overstretching signal at $\sim$65 pN helps to ensure that a specific single target molecule was stretched at the initial experimental test period when probing the force-dependent dynamics of the dimer. Signals observed other than the characteristic DNA overstretching signal should be resulted from the target protein dynamics. Right panel: Two representative force–bead height curves of the α-actinin 1 homo-dimer re-folding and re-dimerization during a linear force-decrease scan with a loading rate of -0.1 pN s^{-1} after rupturing and unfolding during force-increase scans as shown in left panel. For the right panels, the gray down-arrows indicate the re-folding events, and black the down-arrows indicate the re-dimerization events. Here we note that, since the unexpected high mechanical stability of SR full rod dimer that ruptures at $\geq$ 60 pN, similar with the force range of DNA overstretching, we removed the DNA handle in most of our experiments. All data for the figures except this panel was obtained by a configuration without DNA handle. Source data are provided as a Source Data file.

2.6 Supplementary Figure S6. Time-dependent rupture probability analysis of α-actinin rod dimers

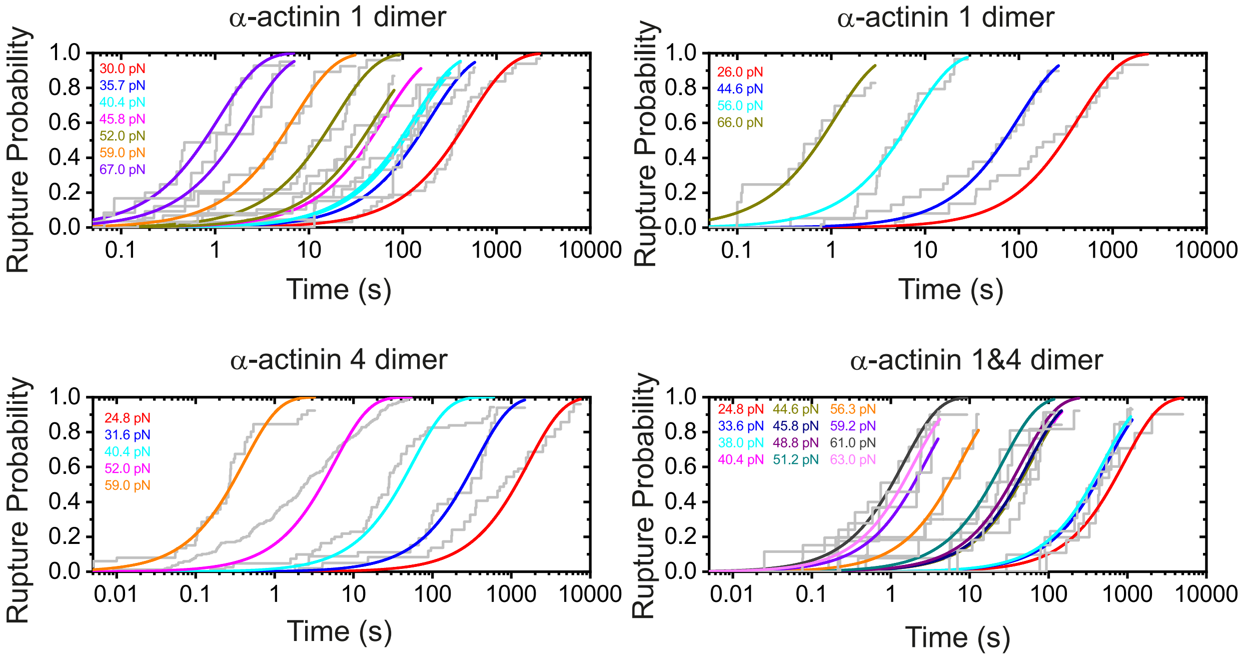

Supplementary Figure S6. Time-dependent rupture probability analysis of α-actinin rod dimers. Time-dependent rupture probability analysis of α-actinin 1 rod homo-dimer (top panels), α-actinin 4 rod homo-dimer (bottom left panel) and α-actinin 1&4 rod hetero-dimer (bottom right panel) under shear-force stretching geometry, respectively. Each light gray line is an everage dependent rupture probability of the dimer obtained from a bootstrap analysis of a data set of lifetimes obtained at the condition.The corresponding colored line is plotted by $P(t) = 1 - \exp(-k_{\text{rupture}} \times t)$, where k_{rupture} value is obtained by fitting to the dependent rupture probability of the dimer using a bootstrap analysis. The forces applied to the dimer were indicated by colors. Source data are provided as a Source Data file.

2.7 Supplementary Figure S7. Time-dependent rupture probability analysis of α-actinin 1 SR2-SR3-SR4:SR1-SR2-SR3 dimer

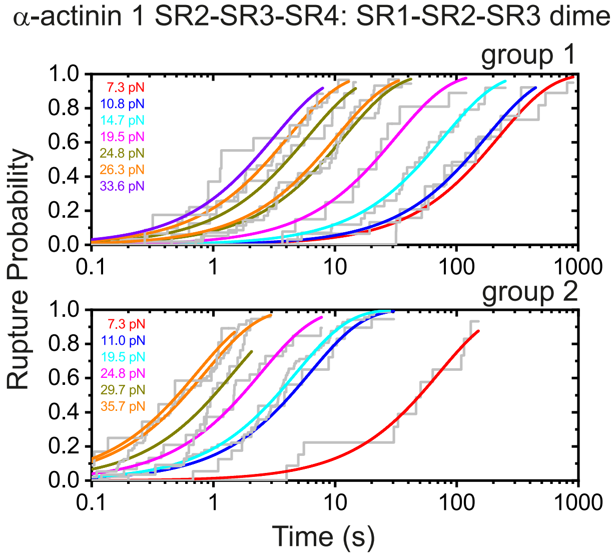

Supplementary Figure S7. Time-dependent rupture probability analysis of α-actinin 1 SR2-SR3-SR4:SR1-SR2-SR3 dimer. Two groups of time-dependent rupture probability analysis of α-actinin 1 SR2-SR3-SR4:SR1-SR2-SR3 dimer under shear-force stretching geometry were obtained and analized. Each light gray line is an everage dependent rupture probability of the dimer obtained from a bootstrap analysis of a data set of lifetimes obtained at the condition. The corresponding colored line is plotted by $P(t) = 1 - \exp(-k_{\text{rupture}} \times t)$, where k_{rupture} value is obtained by fitting to the dependent rupture probability of the dimer using a bootstrap analysis. The forces applied to the dimer were indicated by colors.At each force, lifetimes with distinct distributions were analyzed and plotted separately, e.g., orange lines (26.3 pN) in group 1. Source data are provided as a Source Data file.

2.8 Supplementary Figure S8. Time-dependent rupture probability analysis of *Entamoeba histolytica* α-actinin 2 dimer

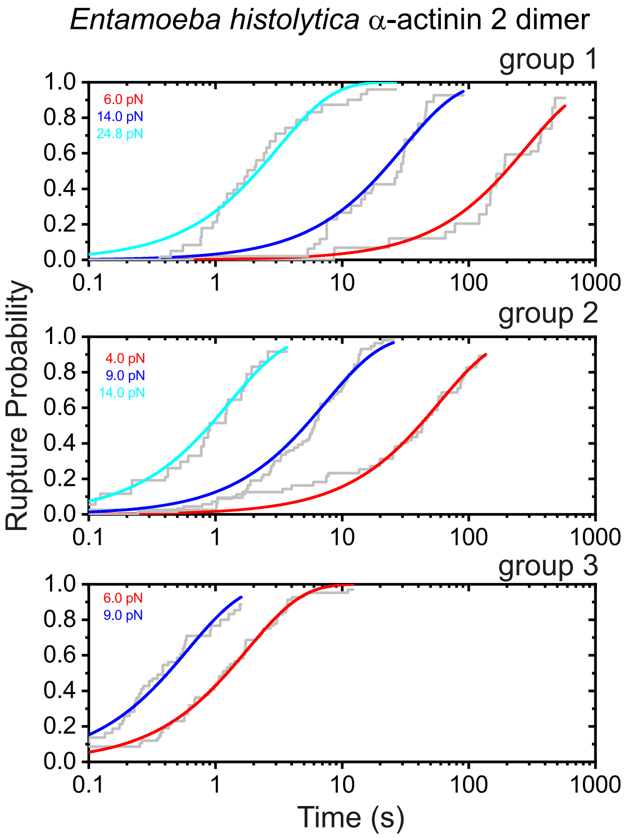

Supplementary Figure S8. Time-dependent rupture probability analysis of *Entamoeba histolytica* α-actinin 2 dimer. Three groups of time-dependent rupture probability analysis of *Entamoeba histolytica* α-actinin 2 dimer under shear-force stretching geometry were obtained and analized. Each light gray line is an everage dependent rupture probability of the dimer obtained from a bootstrap analysis of a data set of lifetimes obtained at the condition.The corresponding colored line is plotted by $P(t) = 1 - \exp(-k_{\text{rupture}} \times t)$, where k_{rupture} value is obtained by fitting to the dependent rupture probability of the dimer using a bootstrap analysis. The forces applied to the dimer were indicated by colors. Source data are provided as a Source Data file.

2.9 Supplementary Figure S9. High mechanical stabiliy of the human α-actinin 1 &4 hetero-dimer under shear-stretching force geometry

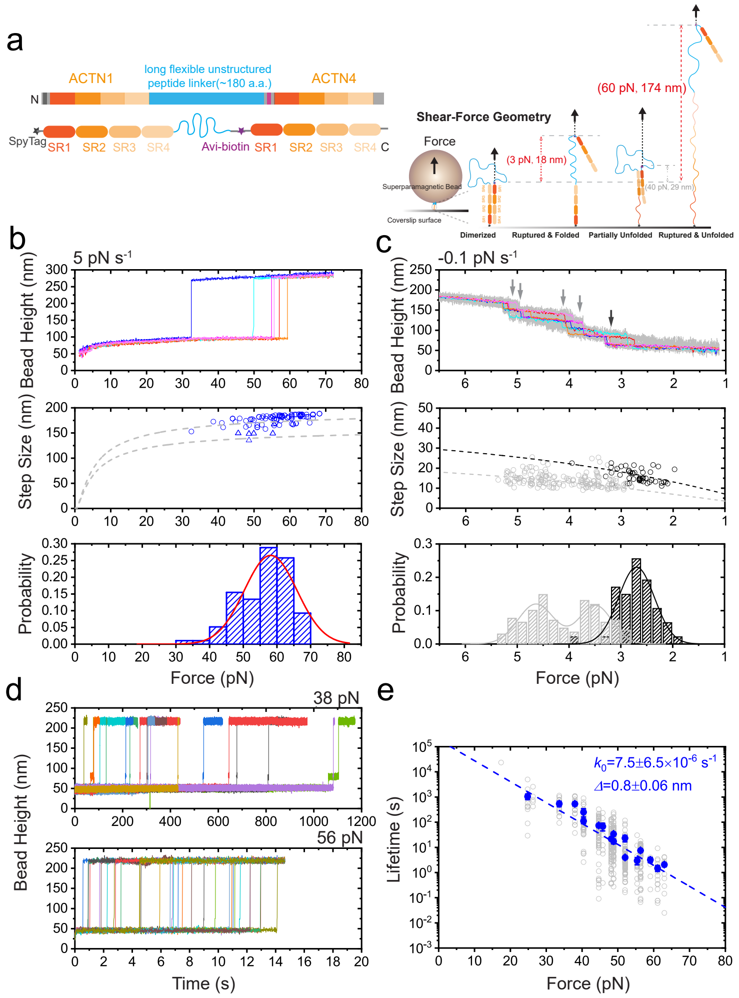

Supplementary Figure S9. High mechanical stabiliy of the human α-actinin 1 & 4 hetero-dimer under shear-stretching force geometry. (a). The illustration of the single-molecule construct and experimental design for quantifying the mechanical stability of the hetero-dimer. **(b&c)**. Top panels: The representative force–bead height curves of α-actinin 1&4 rod hetero-dimer under shear-stretching force geometry during force-increase scans (5 pN s^{-1}, left) and force-decrease scans (-0.1 pN s^{-1}, right), respectively. The gray arrows indicate the refolding events of the SR domains, and the dark gray arrow indicates the re-dimerization event. The light gray lines indicate the raw data obtained during force-loading scan and the colored lines were 10-point FFT smoothing of the raw data. Middle panels: The resulting force–step size graph of the dimer during force-increase scans and force-decrease scans. Left middel panel: the dark gray and gray dashed lines are the theoretically predicated force-dependent stepsizes assuming the cocurrent rupturing and unfolding of all the four SR domains (dark gray) or with three SR domains (gray), respectively. N=97. Right middel panel: the dark gray and gray dashed lines are the theoretically predicated force-dependent stepsizes of the dimer formed by fully folded SR rods (dark gray) and individual SR domains (gray), respectively. re-dimerization events: N=47; re-folding events: N=169. Bottom panels: the normilized rupture forces distribution obtained during force-increase scans with 5 pN s^{-1} (left), and the re-dimerization forces distribution (dark gray) and refolding force distribution (gray) obtained during force-decrease scans with -0.1 pN s^{-1} (right). The lines are gaussian fitting curves of the distributions. **(d)**. The colored lines are representative time–bead height curves of the dimer at two example target forces (38 pN and 56 pN, respectively) obtained by force-jump cycle procedures. The rupture event was indicated by the sudden large stepwize bead height jump at each scan. **(e)**. The force-dependent lifetime of the human α-actinin 1&4 hetero-dimer under shear-stretching force geometry. The dwell times of the dimer at each force were plotted in gray hollow symbols, and the characteristic lifetime of each force was plotted in solid symbols. The blue line is Bell model fitting curve of the blue data sets, and gives k_0 and Δ indicated on the panel. Source data are provided as a Source Data file.

2.10 Supplementary Figure S10. Interconversion of the mechanical states of the α-actinin 1 SR2-SR3-SR4:SR1-SR2-SR3 dimer at a constant force

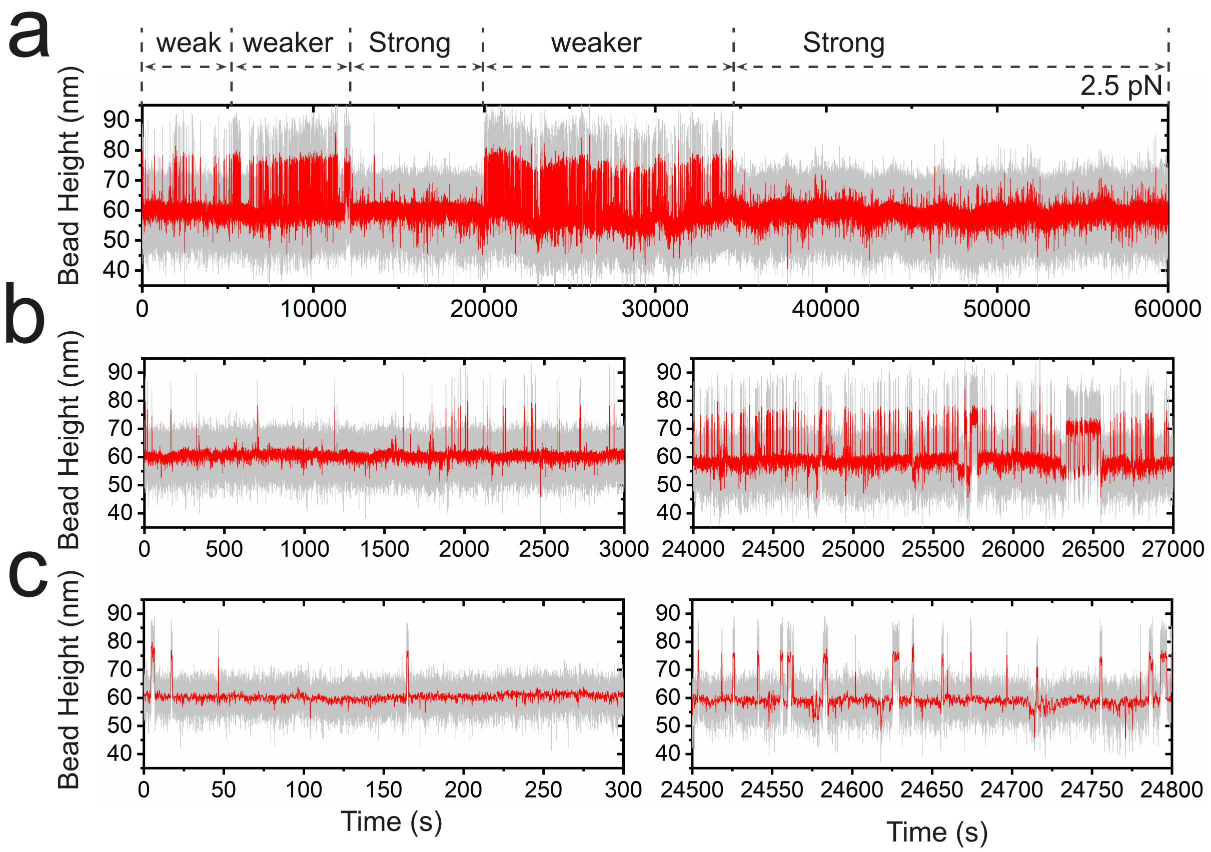

Supplementary Figure S10. Interconversion of the mechanical states of the α-actinin 1 SR2-SR3-SR4:SR1-SR2-SR3 dimer at a constant force. (a). A representative time–bead height curve of the α-actinin 1 SR2-SR3-SR4:SR1-SR2-SR3 dimer at a constant force of ∼2.5 pN over 60000 second recording. **(b)**. Two representative zoom-in time–bead height curves of the dimer at a time scale of 3000 seconds. **(c)**. Two representative zoom-in time–bead height curves of the dimer at a time scale of 300 seconds. The arrows on top panel roughly indicate the time range of three possible mechanical states. Source data are provided as a Source Data file.

2.11 Supplementary Figure S11. Force-response of α-actinin 1 SR2-SR3-SR4 domains

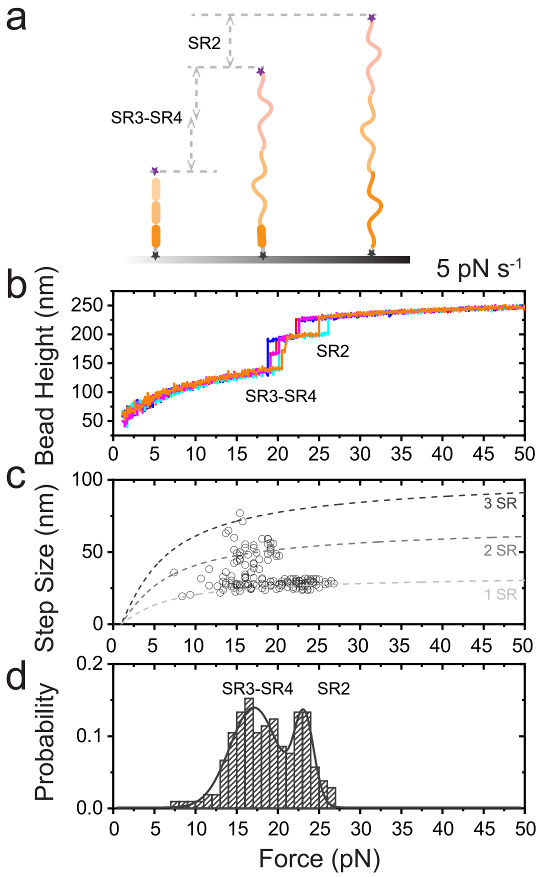

Supplementary Figure S11. Force-response of α-actinin 1 SR2-SR3-SR4 domains. **(a)**. Illustration of the bead height changes of the α-actinin 1 SR2-SR3-SR4 domains unfolding under force. **(b)**. Representative force–bead height curves of the α-actinin 1 SR2-SR3-SR4 domains during force-increase scans at a loading rate of 5 pN s^{-1}. **(c)**. The resulting force–step size graph of the domains unfolding during force-increase scans. The dark gray, gray and light gray dashed lines are the theoretically predicated force-dependent stepsizes assuming the cocurrent unfolding of three, two and one SR domains,respectively. N=150. **(d)**. The normilized unfolding force distribution obtained during force-increase scans with 5 pN s^{-1}. The lines are gaussian fitting curves of the distributions. Source data are provided as a Source Data file.

2.12 Supplementary Figure S12. The representative force–bead height curves of the α-actinin 1 SR1:SR4 pair

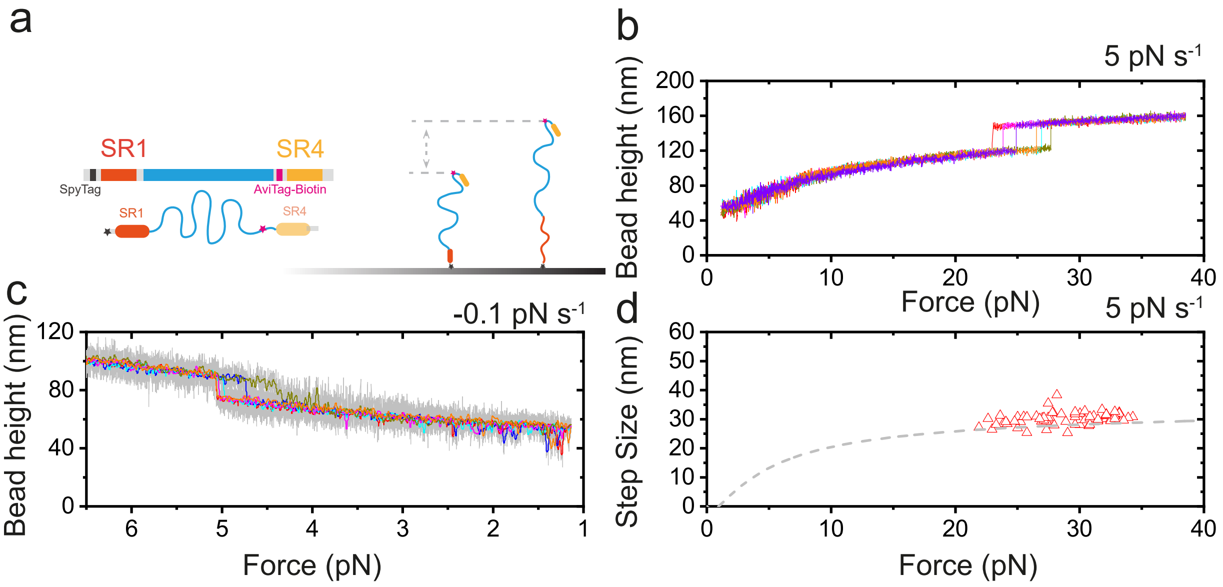

Supplementary Figure S12. The representative force–bead height curves of the α-actinin 1 SR1:SR4 pair.(a).An illustration of the single-molecule design to probe the force response of α-actinin 1 SR1:SR4 pair. **(b&c)** Representative force–bead height curve of the α-actinin 1 SR1:SR4 pair during force-increase scans (5 pN s^{-1}) and force-decrease scans (-0.1 pN s^{-1}).**(d)**. The resulting force–step size curve of the SR1:SR4 pair. Here we note that only SR1 unfolding event was observed. Source data are provided as a Source Data file.

3 Supplementary References

References

[1] Fu, H., Chen, H., Marko, J. F. & Yan, J. Two distinct overstretched dna states. *Biophysical Journal* **100**, 176a (2011).

[2] Zhang, X. *et al.* Revealing the competition between peeled ssdna, melting bubbles, and s-dna during dna overstretching by single-molecule calorimetry. *Proceedings of the National Academy of Sciences* **110**, 3865–3870 (2013).

[3] Zhao, X., Zeng, X., Lu, C. & Yan, J. Studying the mechanical responses of proteins using magnetic tweezers. *Nanotechnology* **28**, 414002 (2017).
