# Supplementary material for: Multi-domain interaction mediated strength-building in human α-actinin dimers unveiled by direct single-molecule quantification
Source: Nat Commun. 2024 Jul 21;15:6151. doi: 10.1038/s41467-024-50430-w (PMC11271494; doi:10.1038/s41467-024-50430-w)
Supplement: Supplementary file 3 — Reporting Summary [file 41467_2024_50430_MOESM3_ESM.pdf]

nature portfolio

Corresponding author(s): Hu Chen, Miao Yu, Shimin Le

Last updated by author(s): Jun 21, 2024

Reporting Summary

Nature Portfolio wishes to improve the reproducibility of the work that we publish. This form provides structure for consistency and transparency in reporting. For further information on Nature Portfolio policies, see our Editorial Policies and the Editorial Policy Checklist.

Statistics

For all statistical analyses, confirm that the following items are present in the figure legend, table legend, main text, or Methods section.

n/a	Confirmed	
☐	☒	The exact sample size (n) for each experimental group/condition, given as a discrete number and unit of measurement
☐	☒	A statement on whether measurements were taken from distinct samples or whether the same sample was measured repeatedly
☒	☐	The statistical test(s) used AND whether they are one- or two-sided<br>*Only common tests should be described solely by name; describe more complex techniques in the Methods section.*
☒	☐	A description of all covariates tested
☒	☐	A description of any assumptions or corrections, such as tests of normality and adjustment for multiple comparisons
☐	☒	A full description of the statistical parameters including central tendency (e.g. means) or other basic estimates (e.g. regression coefficient) AND variation (e.g. standard deviation) or associated estimates of uncertainty (e.g. confidence intervals)
☒	☐	For null hypothesis testing, the test statistic (e.g. F, t, r) with confidence intervals, effect sizes, degrees of freedom and P value noted<br>*Give P values as exact values whenever suitable.*
☒	☐	For Bayesian analysis, information on the choice of priors and Markov chain Monte Carlo settings
☒	☐	For hierarchical and complex designs, identification of the appropriate level for tests and full reporting of outcomes
☒	☐	Estimates of effect sizes (e.g. Cohen's d, Pearson's r), indicating how they were calculated

Our web collection on statistics for biologists contains articles on many of the points above.

Software and code

Policy information about availability of computer code

Data collection	Single protein manipulation data were collected by NI LabVIEW2019.
Data analysis	Single protein manipulation data were analyzed by MATLAB R2021 and OriginPro2021 or OriginPro Learning Edition. Figure panels were plotted by OriginPro2021 or OriginPro Learning Edition, and assembled by Adobe Illustrator 2022.

For manuscripts utilizing custom algorithms or software that are central to the research but not yet described in published literature, software must be made available to editors and reviewers. We strongly encourage code deposition in a community repository (e.g. GitHub). See the Nature Portfolio guidelines for submitting code & software for further information.

Data

Policy information about availability of data

All manuscripts must include a data availability statement. This statement should provide the following information, where applicable:

- Accession codes, unique identifiers, or web links for publicly available datasets
- A description of any restrictions on data availability
- For clinical datasets or third party data, please ensure that the statement adheres to our policy

Key plasmids and protein constructs information is included in supplementary information. Key plasmids are available upon request from the corresponding authors. Source data are provided with this paper.

Research involving human participants, their data, or biological material

Policy information about studies with human participants or human data. See also policy information about sex, gender (identity/presentation), and sexual orientation and race, ethnicity and racism.

Reporting on sex and gender	N.A.
Reporting on race, ethnicity, or other socially relevant groupings	N.A.
Population characteristics	N.A.
Recruitment	N.A.
Ethics oversight	N.A.

Note that full information on the approval of the study protocol must also be provided in the manuscript.

Field-specific reporting

Please select the one below that is the best fit for your research. If you are not sure, read the appropriate sections before making your selection.

☒ Life sciences ☐ Behavioural & social sciences ☐ Ecological, evolutionary & environmental sciences

For a reference copy of the document with all sections, see nature.com/documents/nr-reporting-summary-flat.pdf

Life sciences study design

All studies must disclose on these points even when the disclosure is negative.

Sample size	Sample sizes were determined based on published studies in the field or our previous experiences. No statistics was used to predetermine the sample size. Briefly, in the field of single-molecule manipulation studies, over three individual single-molecules will be manipulated for each experimental procedure. For each individual single-molecule, over 10 repeating cycles of each experimental procedure will be performed. The obtained data points for each experimental procedure are sufficient to show characterized distributions, which can be well fitted with theoretical models.
Data exclusions	No data were excluded.
Replication	Each experiment was performed on multiple independent molecule tethers (>3) .
Randomization	No sample randomization was performed.
Blinding	The investigators were not blinded for group allocation.

Reporting for specific materials, systems and methods

We require information from authors about some types of materials, experimental systems and methods used in many studies. Here, indicate whether each material, system or method listed is relevant to your study. If you are not sure if a list item applies to your research, read the appropriate section before selecting a response.

Materials & experimental systems

n/a	Involved in the study	
☒	☐	Antibodies
☒	☐	Eukaryotic cell lines
☒	☐	Palaeontology and archaeology
☒	☐	Animals and other organisms
☒	☐	Clinical data
☒	☐	Dual use research of concern
☒	☐	Plants

Methods

n/a	Involved in the study	
☒	☐	ChIP-seq
☒	☐	Flow cytometry
☒	☐	MRI-based neuroimaging

Plants

Seed stocks	N.A.
Novel plant genotypes	N.A.
Authentication	N.A.
